# Genetic diversity for agromorphological traits, phytochemical profile, and antioxidant activity in Moroccan sorghum ecotypes

**DOI:** 10.1038/s41598-022-09810-9

**Published:** 2022-04-07

**Authors:** Youssef Bouargalne, Reda Ben Mrid, Najat Bouchmaa, Zakia Zouaoui, Bouchra Benmrid, Anass Kchikich, Redouane El Omari, Imad Kabach, Nhiri Mohamed

**Affiliations:** 1 Laboratory of Biochemistry and Molecular Genetics, Faculty of Science and Technology of Tangier, Abdelmalek Essaâdi University, BP 416, 90000 Tangier, Morocco; 2 Institute of Biological Sciences (ISSB-P), Mohammed VI Polytechnic University (UM6P), 43150 Ben-Guerir, Morocco; 3 Team of Experimental Oncology and Natural Substances, Cellular and Molecular Immuno-Pharmacology, Faculty of Science and Technology, Sultan Moulay Slimane University, Beni-Mellal, Morocco; 4 AgroBioSciences Research Division, Mohammed VI Polytechnic University, Lot 660–Hay Moulay Rachid, 43150 Ben-Guerir, Morocco; 5 Higher School of Technology (EST) Sidi Bennour, Chouaib Doukkali University, El Jadida, Morocco

**Keywords:** Biochemistry, Biotechnology, Plant sciences, Health care

## Abstract

Sorghum, the fifth most important cereal crop, is a well-adapted cereal to arid/semi-arid regions. Sorghum is known for multiple end-uses as food, feed, fuel, forage, and as source of bioactive compounds that could be used for medical applications. Although the great improvement in the process of sorghum breeding, the average yield of this crop is still very low. Therefore, exploring the genetic diversity in sorghum accessions is a critical step for improving this crop. The main objective of the current work was to study the genetic variation existing in a Moroccan sorghum collection. Indeed, 10 sorghum ecotypes were characterized based on agromorphological descriptors. Both quantitative (25) and qualitative (7) traits revealed variability (*p* < 0.05) among the studied ecotypes. At the seedling stage, most of the ecotypes showed good to high vigor (70%). However, as the sorghum plants grow, the difference between genotypes become more apparent, especially at the generative phase. For instance, three different panicle shapes have been observed, erect (50%), semi-bent (30%), and bent (20%) with different degree of compactness (20% for loose, semi-compact, and compact panicles, and 30% for semi-loose panicles). In another part of this study, the phytochemical composition and antioxidant activities of the sorghum ecotypes have been determined. The results showed variable total phenolic contents, and total flavonoid contents ranging from 125.86 ± 1.36 to 314.91 ± 3.60 mg GAE/g dw and 114.0 ± 13.2 to 138.5 ± 10.8 (mg catechin equivalent/100 g, dw) respectively, with a differential antioxidant activities as well. These results indicate that for any crop breeding program, it is preferable to take into consideration both morphological and biochemical traits for a better selection of high yielding varieties with high added value compounds. Therefore, the implication of these results in the context of sorghum breeding activities could be a resourceful option for farmers.

## Introduction

Sorghum (*Sorghum bicolor* (L.) Moench; 2n = 2x = 20; family Poaceae), a cereal crop originated in Sub-Saharan Africa. It ranks fifth among top-five crops in terms of production and consumption in the world and fourth among cereals in Africa in terms of total production (> 25 M tons) where it is mainly used as human food^1^. Sorghum is a C4 crop of great socio-economic and ecological importance for many countries, especially in arid and semi-arid regions where it plays an important role in attaining food security. Sorghum has been utilized for multiple purposes, for instance, as food in many parts of Africa and Asia, and as animal feed in the western region of Africa^2^. Regarding their nutrition value, sorghum grains are not much different from other cereals. They are mainly composed of starch, protein, non-starch polysaccharides, and fatty acids. However, these grains do not contain gluten^3^, which make them a good choice of food for people who have gluten intolerance.

In addition to be a human food resource, sorghum grains are also used in traditional medicine in Africa and Asia^4,5^. Additionally, previous studies have reported the ability of grain extracts of different sorghum varieties to exhibit several biological activities such as antioxidant activities^2,6,7^. This antioxidant activity is itself correlated to the high contents of sorghum grains in bioactive compounds. Indeed, it was reported that sorghum grains contain the highest phytochemical levels among cereals such as flavonoids, phenolic acids and anthocyanins^2,8,9^. Polyphenols from sorghum, such as proanthocyanidin and 3-deoxyanthocyanidin were reported to be endowed with multiple health benefits and might ensure protection against oxidative stress, inflammation and diabetes. Flavonoids, on the other hand, are well known for their antioxidant activity and their ability to act as signaling molecules and several reports correlated their consumption with lower incidence of different diseases such as cancer, diabetes, inflammatory diseases, and neurodegenerative diseases^10–12^. These compounds were reported to vary between sorghum genotypes^9^, which led us to suggest that these traits could help breeders to select varieties of sorghum plants with the highest added health benefits and to combine these traits to agronomic traits for a final goal of selecting high yield sorghum varieties endowed with high beneficial health effects. Indeed, sorghum is considered as a minor cereal in Morocco, and it is cultivated on 29,000 ha with a production that never exceeds 9 quintals/ha^13^. This cereal crop is traditionally grown, mainly, in the northwestern regions of Morocco at an altitude varying between 20 and 800 m^13^. The identification, characterization, exploration, and the collection of local sorghum genotypes is considered as the first step in order to preserve and develop genetic resources and increase the genetic quality of sorghum varieties through plant breeding programs^14^.

Analysis of the germplasm lines for morphological and biochemical diversity is desired before further utilization of these lines in conventional or molecular breeding programs. A high degree of morphological variation among landraces, which were assigned to the race durra, bicolor, and their intermediates, was previously discussed^13^. It was reported by Naoura et al.^15^ that PCA and clustering analysis of morphological characterization, are relatively inexpensive and easy to carry out (1) for conservation of genetic resources, (2) identification of characters amenable to genetic improvement, and (3) selection of high yielding sorghum genotypes. Therefore, the objective of the present study was to analyze the variability and determine relationship between morphological (18 quantitative and 7 qualitative) and biochemical traits (total phenolic content, flavonoid content, and antioxidant activity) among 10 Moroccan sorghum ecotypes. These last were collected from the North Western region of Morocco and used after three cycles of selection in order to homogenize the plant material, while maintaining the genetic diversity. A preliminary experiment was then conducted for the different ecotypes and allowed us to select 10 ecotypes which exhibited high homogeneity in different phenotypic traits within replicates of the same ecotype while maintaining a remarkable genetic diversity between the different ecotypes.

Findings from the present investigation will help identify high-performance ecotypes for key agronomic traits for utilization in future breeding programs that targets the selection of high yielding cultivars endowed with good nutritional and biological quality.

## Results and discussion

### Morphological variation

In the present study, qualitative and quantitative agromorphological traits of the ten studied sorghum ecotypes were recorded. Of note, qualitative traits are preferred by farmers and used to name and identify distinct sorghum cultivars^16^. In our study, a significant genetic variation among ecotypes was noticed (Tables 1, 2). In fact, 70% of the seedlings were found to have good to high vigor, while 10% were rated as having bad vigor. The difference between sorghum genotypes can be identified more clearly in the generative phase (from the development of flowers to seed ripening)^17^. In the vegetative stage the plant height of the tested ecotype reached 179 cm with an EX that varied from 25 to 63 cm. In fact, the variation was more pronounced in PL as it reached around 25 (minimum) to 63 cm (maximum) (SD = 9.1 cm). The width and length of flag leaf ranged from 3.00 to 5.80 and 30.00 to 46.00 cm, respectively. Whereas the diameter and length of the third internode, WTF, and LTF from the top showed a slight variation, not of much significance. Furthermore, the role of leaves in producing dry matter during the photosynthesis process is crucial for optimum yield^18^. In our study, there were three phenotypic classes observed for panicle shape, however, the erect type was the dominant type (50%). Nonetheless, for panicle compactness, a high frequency was recorded for semi-loose type (30%). Whereas the other characters (loose, semi-compact, and compact) showed a decreased frequency (20%). Out of the wide variability observed in this study, Chantereau et al.^19^ stated that dry-season sorghum has reduced tillering, compact panicles, and an often curved peduncle. These traits are preferred by farmers as it facilitates protection against bird attacks, as well as for their big grains with vitreous albumen. Likewise, Naoura et al.^15^ showed that dry-season sorghum cultivars had compact panicles, glume hairiness, and black glumes.

**Table 1:** Frequency (%) of different phenotypic classes for 7 qualitative traits in the established collection of Moroccan sorghum.

Traits	Modality	Frequency (%)
Seedling vigor (GV)	Bad vigor	10
	Weak vigor	20
	Good Vigor	20
	Very good vigor	20
	High vigor	30
Peduncle shape (PS)	Erect	50
	Semi-bent	30
	Bent	20
Panicle compactness (PC)	Loose	20
	Semi-loose	30
	Semi-compact	20
	Compact	20
Glume covering (GCOV)	25% grain covered	10
	50% grain covered	40
	75% grain covered	40
	100% grain covered	10
Glume color (GLC)	Beige	20
	Brown	60
	Purple	20
Grain color (GCOL)	White	10
	Beige	10
	light Brown	40
	Brown	40
Grain shape (GSH)	Rounded	40
	Oval	50
	Elliptic	10

**Table 2:** Descriptive statistics of 14 quantitative traits in 10 Moroccan sorghum ecotypes.

	Min	Max	Mean ± SE	SD	1st Qu	Median	3rd Qu
PH	98.00	179.00	131.79 ± 3.34	25.86	108.50	125.00	154.20
LFL	17.50	31.00	23.06 ± 0.42	3.27	20.93	22.00	25.00
WFL	2.40	6.80	3.99 ± 0.15	1.13	3.25	3.85	4.50
LTF	30.00	46.00	37.77 ± 0.57	4.45	35.00	36.25	40.50
WTF	3.00	5.80	4.47 ± 0.09	0.67	4.00	4.45	5.00
NI	5.00	9.00	6.20 ± 0.14	1.09	6.00	6.00	6.00
DTI	0.40	1.40	0.96 ± 0.03	0.25	0.80	1.00	1.13
LTI	8.70	19.00	12.46 ± 0.28	2.20	11.07	12.00	13.62
PL	25.00	63.00	41.21 ± 1.18	9.11	34.00	40.50	49.50
EX	7.50	35.00	19.35 ± 1.06	8.21	14.00	17.00	26.00
PANL	6.50	25.00	14.20 ± 0.55	4.30	11.50	14.00	17.00
PW	3.20	12.00	7.33 ± 0.30	2.36	5.50	7.50	8.63
NPBP	6.00	16.00	10.38 ± 0.31	2.37	9.00	10.50	11.00
HGW	1.09	3.22	2.34 ± 0.06	0.47	1.98	2.33	2.61
DFL	32.00	96.00	57.17 ± 2.35	18.20	40.00	55.00	72.00
NDF	61.00	139.00	89.07 ± 2.80	21.71	70.75	87.00	99.50
GP	84.00	100.00	92.05 ± 0.67	5.20	87.75	91.50	97.00

*PH* Plant height (cm), *LFL* length of flag leaf, *WFL* width of flag leaf, *LTF* length of third leave, *WTF* width of third leave, *NI* number of internodes, *DTI* diameter of third internode (cm), *LTI* Length of Third internode (cm), *PL* Peduncle length (cm), *EX* Peduncle exertion (cm), *PANL* Panicle length (cm), *PW* panicle width (cm), *NPBP* number of primary branches per panicle, *HGW* Weight of 100 grains (g), *DFL* Date to flag leaf appearance, *NDF* Number of days at flowering (days), *GP* Germination percentage %.

Regarding NDF and DFL of the ten studied ecotypes, results showed the presence of early and late-maturing cultivars in our collection which is in line with the classification of early, mid and late-maturing varieties of sorghum given by Tabri and Zubachtirodin^20^. The variation in NDF and DFL also suggests the difference in the duration of grain filling^21^. This is supposed to contribute to the grain size (HGW) variation from 1.09 to 3.22 g of 100 grains observed in the ecotypes. The difference stated above regarding PL, EX, NDF, and DFL, might be accounted for much of the difference in HGW which varied between 1.09 and 3.22 g. Glume color and grain color are also important morphological traits that allow us to differentiate between different sorghum cultivars^22^. At maturity, 50–75% of the grains in our experiments were found to be covered with glumes. In line with this, results by Thakur et al.^23^ reported that glumes coverage seems to be an adaptive feature that minimizes grain mold. In addition, most cultivars had brown glumes (60%) with an oval shape (50%). These results are in accordance with the results obtained by Ayana and Bekele^20^ where compact and oval grains were the dominant characters among all of the studied sorghum landraces. On the other hand, results from our study revealed that the majority of the grains had different color frequencies with dark color being the most dominant (40% light brown, 40% brown). Grain color has been reported to be associated with grain quality as stated by several farmers in the study of Diallo et al.^24^. Indeed, they affirmed that white grains are more preferred to be sold as food, while they associated dark color grains with animal feed^24^. More interestingly, Nazari et al.^25^ stated that grain color could also be an indicator of the phenolic compounds content which is, in turn, a determinant indicator of sorghum nutritional quality. In fact, these authors declared that red sorghum varieties had higher condensed tannin contents compared to white ones. In this regard, it was reported that the presence of condensed tannin could have a considerable agronomic advantage for sorghum crops as it protects them from bird predation, as well as from insect and fungal attacks. However, a high content of these compounds was found to be associated with reduced nutritional quality of sorghum^26^. Seeds from our collection are considered to be of good quality with high physiological potential owing to their higher germination percentage (80–100%). In fact, the germination percentage appears to be a potential indicator for breeders to screen cultivars as stated by before^27^. Taken together, a higher variation for different characteristics/traits in our collection indicated a greater ability for its improvement through selection^28^.

### Correlation analysis among agromorphological traits

The correlation analysis is used as an indicator to facilitate the selection of promising ecotypes that combine different agronomically important traits. In this study, Pearson correlation coefficients revealed significant associations between the different quantitative traits evaluated (Table 3). The PH had a significant positive correlation with LFL and DTI (*r* = 0.373, *P* = 0.039) and a highly significant positive correlation with PL (*r* = 0.428, *P* = 0.000), EX (*r* = 0.630, *P* = 0.000), GP, PW and PANL (*r* = 0.556, *P* = 0.000), but an unexpected, negative, highly significant correlation with NI (*r* = − 0.295, *P* = 0.000), LTI and HGW (*r* = − 0.391, *P* = 0.009). Similar to our results, Naoura et al.^15^ showed a significant correlation among a few quantitative parameters evaluated. Indeed, they revealed that the potential yield was positively influenced by length of the penultimate leaf, penultimate leaf width, number of green leaves and the weight of the main panicle. As unexpected, the yield potential negatively correlated with longer peduncle length and penultimate leaf sheath length.

**Table 3:** The correlation coefficient of quantitative traits evaluated in 10 Moroccan sorghum ecotypes.

	PH	LFL	WFL	LTF	WTF	NI	DTI	LTI	PL	EX	PANL	PW	NPBP	HGW	DFL	NDF	GP
PH	1.000																
LFL	0.216*	1.000															
WFL	− 0.125*	− 0.366***	1.000														
LTF	0.161	0.714***	− 0.172***	1.000													
WTF	− 0.102	0.512**	− 0.268**	0.412**	1.000												
NI	− 0.295***	− 0.259*	0.201	0.040	0.243	1.000											
DTI	0.373*	0.522***	− 0.203***	0.376**	0.659**	0.048	1.000										
LTI	− 0.092***	0.478**	− 0.259**	0.579***	0.445**	− 0.015	0.071	1.000									
PL	0.428***	0.504**	− 0.154*	0.163	0.042	− 0.604***	0.348*	0.075	1.000								
EX	0.630***	0.310	0.002	0.067	− 0.078	− 0.411***	0.325	0.016	0.818***	1.000							
PANL	0.556***	0.567***	0.032*	0.485*	0.227	− 0.382***	0.579**	0.233	0.697***	0.705***	1.000						
PW	0.469***	0.710***	− 0.128**	0.483**	0.346	− 0.417***	0.652***	0.185	0.771***	0.669***	0.843***	1.000					
NPBP	0.190	− 0.053	0.435*	− 0.189**	0.248	− 0.175	0.376	− 0.030*	0.235	0.371	0.441	0.304	1.000				
HGW	− 0.391**	− 0.459***	0.779***	− 0.321***	− 0.341*	0.302*	− 0.332***	− 0.355**	− 0.206**	− 0.077	− 0.163**	− 0.236***	0.294*	1.000			
DFL	− 0.118*	− 0.263**	0.585***	− 0.224***	0.157	0.109	0.039*	0.088	− 0.113*	0.095	0.125*	− 0.045**	0.846***	0.534***	1.000		
NDF	− 0.089*	− 0.310***	0.590***	− 0.259***	0.172	0.259	0.093*	0.059	− 0.170**	0.085	0.074*	− 0.121**	0.823***	0.524***	0.960***	1.000	
GP	0.548**	0.165	0.065	0.010	− 0.289*	− 0.512***	0.109	0.043	0.641***	0.796***	0.641***	0.541**	0.352	0.005	0.139	0.104	1.000

*PH* Plant height (cm), *LFL* length of flag leaf, *WFL* width of flag leaf, *LTF* length of third leave, *WTF* width of third leave, *NI* number of internodes, *DTI* diameter of third internode (cm), *LTI* Length of Third internode (cm), *PL* Peduncle length (cm), *EX* Peduncle exertion (cm), *PANL* Panicle length (cm), *PW* panicle width (cm), *NPBP* number of primary branches per panicle, *HGW* Weight of 100 grains (g), *DFL* Date to flag leaf appearance, *NDF* Number of days at flowering (days), *GP* Germination percentage %

Significance: *P < 0.05; **P < 0.01; ***P < 0.001.

According to several published literature, flag leaves are important for wheat plants photosynthetic performance. Indeed, flag leaf was suggested to be an important determinant of plant architecture and yield potential, thus, a good trait to be targeted for breeding and cultivar development in wheat^29,30^. In this context, results from our experiments revealed that the width of flag leaf exhibited a highly significant positive correlation with NPBP, HGW, DFL, and NDF. However, it showed a highly negative correlation with LTF, WTF, DTI, LTI and PW. These results suggest that the optimal flag leaf morphology is an important character that improves light absorption, which in turn improves photosynthesis and might eventually lead to higher grain yield potential. Furthermore, an unexpected negative correlation was found between WFL and LFL suggesting that yield increases were achieved through the selection of a smaller leaf size. Moreover, GP exhibited a highly positive and significant correlation with panicle quantitative traits (PL, EX, PANL, and PW) and PH (*r* = 0.584, *P* = 0.009), while it showed a negatively significant correlation with NI (*r* = − 0.512, *P* = 0.000). This result indicates that the selection of plants with greater NI may decrease their GP at the beginning of the crop cycle. The positive and significant association between grain yield and DFL, NDF, and WFL indicated that these traits are crucial for higher sorghum yields. Therefore, the selection of any one of these yield attributing traits will lead to an increase in the other traits, subsequently enhancing the overall grain yield. Vegetative traits such like, PH, and LTF can be prioritized in the selection of an ecotype with higher grain yield considering strong negative correlation observed between them. This negative correlation should be explained by the time taken by plants to accumulate biomass^31^. Moreover, grain yield is a complex character which is highly influenced by the environment and is the result of interrelationship of its various yield components^32^.

### Principal component analysis (PCA) of various quantitative traits

Factor analysis was performed in order to eliminate redundancy in the data variability because genetic improvement depends on the magnitude of genetic variation^33^. Five principal components (Dim.1 to Dim.5) having eigenvalues of > 1 were extracted (Table 4). These principal components accounted for most of the variability observed among the sorghum accessions collected from the different locations (86.01% of total variance). The Dim.1 contributed to 33.76% of the agromorphological variation followed by Dim.2 (23.20%), Dim.3 (15.83%), Dim.4 (7.02%), and Dim.5 (6.2%). Dim.1 was loaded on PH (62.3% of variation factor), LFL (75.6% of variation factor), LTF, WTF (32% of variation factor), DTI, LTI, PL, EX, PANL, PW and GP (36.9% of variation factor), NI (33.4% of variation factor) and HGW, as these traits registered above 30% variation factor. For the Dim.2, we obtained a high and positive factor with the traits such as WFL (72.9%), EX (37.9%), NPBP (86.2%), HGW (65.5%), DFL (86.9%), NNDF (85.9%), and a negative loading with LFL (33.3%) and LTF (35.7%). Following a similar logic, Dim.3 was positively correlated with LFL (32.3%), LTF (45.6%), WTF (84.4%), NI (51.7%), DTI (46.3%), LTI (52.1%), DFL (36.9%) and NDF (40.1%) and was negatively correlated with PH (32.4%), PL (31.6%), EX (34.9%), and GP (47%). The Dim.4 was negatively correlated with PH (33.4%) and DTI (50.6%) while it showed a positive correlation with LTF (40.7%) and LTI (54.9%). The Dim.5 showed a positive correlation with WFL (40.3%), LTF (31.2%), NI (43.7%), and HGW (39.4%) and a negative loading with LTI (40.3%).

**Table 4:** Principal component analysis (PCA) of different quantitative traits in the 10 Moroccan sorghum ecotypes.

	Dim.1	Dim.2	Dim.3	Dim.4	Dim.5
Eigenvalues	5.740	3.943	2.692	1.193	1.054
% of variance	33.762	23.196	15.833	7.019	6.200
Cumulative % of variance	33.762	56.958	72.791	79.810	86.010
PH	0.623	0.103	− 0.324	− 0.334	0.088
LFL	0.756	− 0.333	0.323	0.205	0.094
WFL	− 0.306	0.729	0.017	0.266	0.403
LTF	0.519	− 0.357	0.456	0.407	0.312
WTF	0.326	− 0.122	0.844	− 0.236	− 0.107
NI	− 0.505	− 0.026	0.517	− 0.151	0.437
DTI	0.639	0.053	0.463	− 0.506	0.210
LTI	0.315	− 0.213	0.521	0.549	− 0.403
PL	0.816	0.136	− 0.316	0.068	− 0.020
EX	0.749	0.379	− 0.349	− 0.015	0.026
PANL	0.876	0.287	0.047	0.100	0.162
PW	0.915	0.103	0.058	0.019	0.196
NPBP	0.256	0.862	0.247	− 0.187	− 0.224
HGW	− 0.474	0.655	− 0.045	0.281	0.394
DFL	− 0.124	0.869	0.369	0.069	− 0.237
NDF	− 0.172	0.859	0.401	− 0.032	− 0.179
GP	0.618	0.420	− 0.470	0.177	− 0.091

*PH* Plant height (cm), *LFL* length of flag leaf, *WFL* width of flag leaf, *LTF* length of third leave, *WTF* width of third leave, *NI* number of internodes, *DTI* diameter of third internode (cm), *LTI* Length of Third internode (cm), *PL* Peduncle length (cm), *EX* Peduncle exertion (cm), *PANL* Panicle length (cm), *PW* panicle width (cm), *NPBP* number of primary branches per panicle, *HGW* Weight of 100 grains (g), *DFL* Date to flag leaf appearance, *NDF* Number of days at flowering (days), *GP* Germination percentage %.

The Dim.2 showed a strong and positive factor with the traits like panicle weight (42.2%) and grain yield (49.5%) and a negative loading with the days to 50% flowering (30.5%). Moreover, Dim.3 negatively correlated with the stem third internode girth (30.1% of variation factor), the panicle width (51.2% of variation factor), and the panicle length (56.1% of variation factor). The Dim.3 showed that diversity among accessions depended on days to 50% seed germination (43.4% of variation factor) and 100-seed weight with negative loadings (36.3% of variation factor). Dim.5 confirms the agromorphological variation observed at Dim.4 by high positive factors. The remaining variable had weak or no discriminatory power. Thus, the most important descriptors were those associated with Dim.1, Dim.2 and Dim.3.

**Figure 1:**
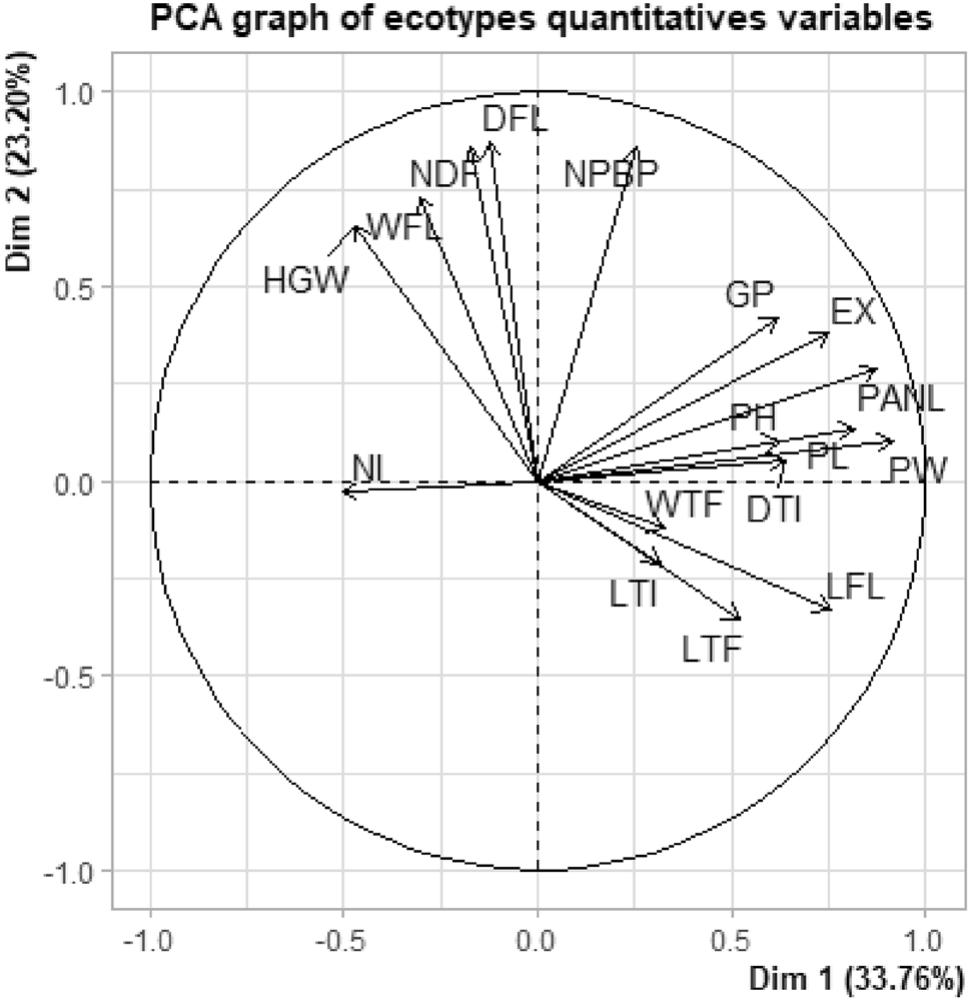
Distribution of quantitative traits in the first two factors (represents 55.96% of the information) of 10 Moroccan sorghum ecotypes. (HGW: Weight of 100 grains (g); WFL: width of flag leaf; NDF: Number of days at flowering (days); DFL: Date to flag leaf appearance; NPBP: number of primary branches per panicle; GP: Germination percentage %; EX: Peduncle exertion (cm); PH: Plant height (cm); PANL: Panicle length (cm); PL: Peduncle length (cm); PW: panicle width (cm); WTF: width of third leave: DTI: diameter of third internode (cm); LFL: length of flag leaf; LTI: Length of Third internode (cm) LTF: length of third leave).

**Figure 2:**
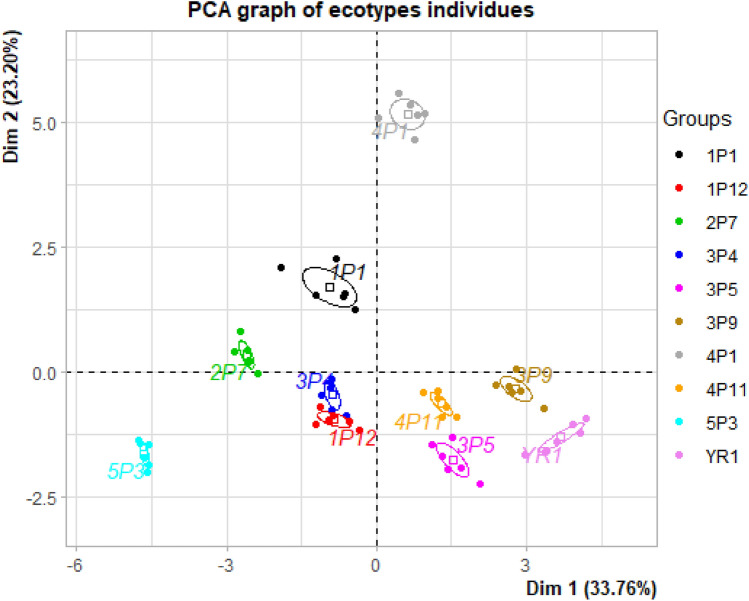
The distribution of sorghum ecotype for the first two principal components (Dim.1 = 33.76% and Dim.2 = 23.20%) based on quantitative traits.

Fenty^34^ reported that PCA reduces a large set of variables to come up with smaller sets of components that summarize the correlations. It is observed that at the loading plot (Fig. 1), the distribution of quantitative traits in the first two factors (represents 55.96% of the information), explained the quantitative traits of Moroccan sorghum ecotypes. Consequently, it can be inferred that the selection of traits WFL, NDF, and DFL may also be given importance along with yield. Figure 2 showed the presence of a significant amount of variability in sorghum ecotypes. These results indicated that PCA analysis is a reliable method in the evaluation of sorghum accessions for crop production. Our results are in line with previous findings by Makanda et al.^35^, who found that significant differences among the different quantitative morphological traits and the first two principal components had the maximum contribution of the genetic diversity among the sorghum accessions^35^. These results were also in agreement with previous findings by Mumtaz et al.^36^ who also reported a highly significant genetic variation among sorghum accessions.

**Figure 3:**
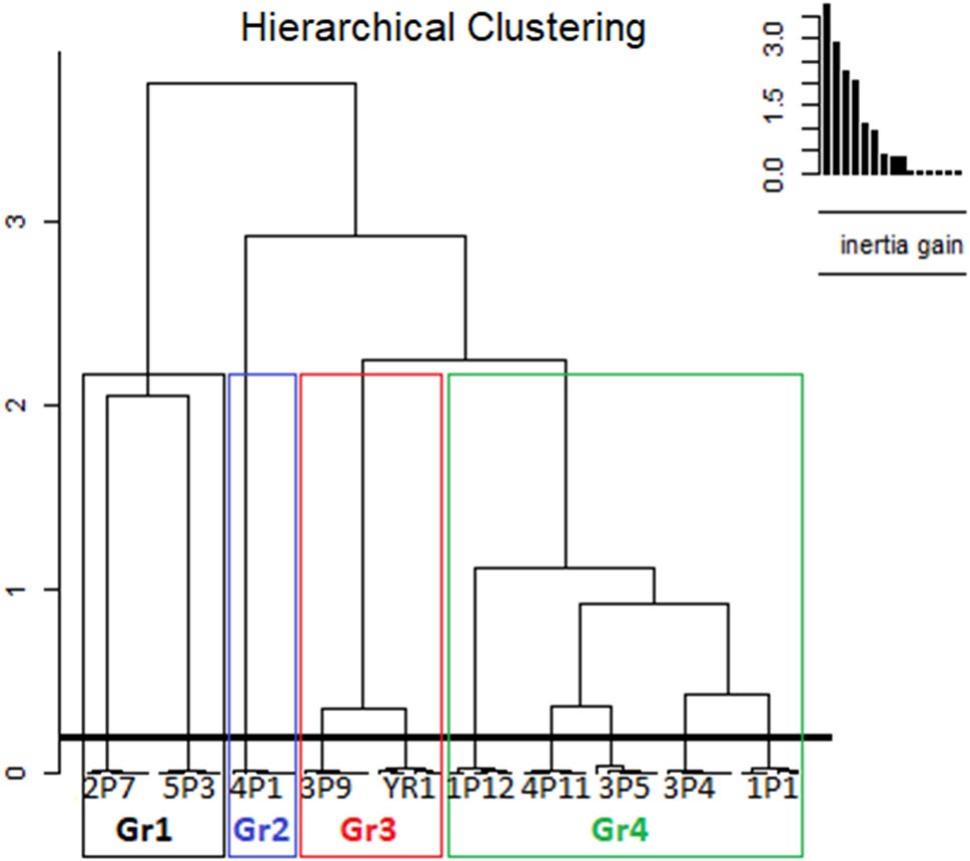
Hierarchical ascendant classification of Moroccan sorghum ecotypes based on agromorphological traits.

The hierarchical clustering analysis of the 10 sorghum accessions based on the combination of both the qualitative and the quantitative morphological traits is given in Fig. 3. Four major groups (Gr) were formed among the sorghum ecotypes. Similarly, Naoura et al.^15^ revealed that, through ten less correlated traits, the morphological cluster analysis allowed the cultivars to be clustered into four statistically distinct groups, with group 2 cultivars showed high productivity, precocity, and the stay-green, which all contributed to better cultivar performance, thus, providing useful suggestions for breeding programs. The number of ecotypes per cluster varied from 1 to 5 ecotypes. Cluster I and III were composed of two ecotypes, 2P7, 5P3 and 3P9, YR1 respectively. Cluster II was represented by only one ecotype (4P1) having compact panicle, greater 100 grain weight, light brown grain color along with a high vigor. The largest cluster unit (Gr4) gathered 5 accessions (1P12, 4P11, 3P5, 3P4, 1P1) with close similarity in vegetative traits and many differences in qualitative traits. In Morocco, the selection is generally made by farmers and they tend to use qualitative traits rather than quantitative traits to make their own seedling stock. In contrary, our results revealed that the clustering pattern of sorghum ecotypes having similarity in quantitative traits and differences in qualitative traits were frequently present in the same cluster. These results suggest that both qualitative and quantitative variables can reveal diversity providing different but complementary information. Therefore, for any selection programs in Morocco, the choice of suitable ecotypes must take into consideration both qualitative and quantitative traits^37^. The clustering pattern indicated the existence of a significant amount of variability among the sorghum ecotypes. The highest inter-cluster distance was observed between the cluster I/II and the cluster IV, suggesting that accessions from these clusters were genetically different. The ecotypes from high distance clusters have a maximum divergence of variability which gives the greater emphasis of breeder to use them for the purpose of the selection process^31^. The ecotype from those clusters can be used as parents in hybridization program and may give the broad spectrum of the variability in the segregating generation^38^.

### Antioxidant activity, total phenolic and flavonoid contents in accessions of Sorghum bicolor (L.) Moench

In our study, total phenolic content, total flavonoid content, and antioxidant activities presented in Table 5 were considered as biochemical traits of the 10 Moroccan Sorghum ecotypes.

**Table 5:** Total phenolic content, flavonoid content, and antioxidant activities in methanolic extracts of 10 Moroccan sorghum ecotypes.

Sorghum ecotypes	Antioxidant properties (IC_50_ Values; mg/mL)			Reducing Power (mg AAE/g dw)	Flavonoids (mg QE/g dw)	Polyphenols (mg GAE/g dw)
	DPPH	ABTs	Metal Chelating Activity			
1P1	0.05 ± 0.00^a^	0.34 ± 0.02^a^	2.37 ± 0.27^a^	103.15 ± 2.81^a^	14.04 ± 1.71^a^	159.23 ± 4.48^a^
1P12	0.02 ± 0.00^b^	0.20 ± 0.00^b^	3.11 ± 0.33^a^	107.19 ± 1.24^a^	7.35 ± 0.16^b^	255.97 ± 6.29^b^
2P7	0.04 ± 0.00^c^	0.28 ± 0.02^c^	2.76 ± 0.15^a^	110.50 ± 3.88^a^	9.96 ± 1.53^cf^	204.49 ± 5.05^c^
3P4	0.04 ± 0.00^c^	0.24 ± 0.02^ cd^	2.47 ± 0.10^a^	112.47 ± 3.65^a^	4.53 ± 0.91^d^	148.71 ± 6.70^d^
3P9	0.02 ± 0.00^b^	0.19 ± 0.00^b^	2.76 ± 0.15^a^	111.74 ± 2.98^a^	13.04 ± 0.67^ae^	277.52 ± 4.10^e^
4P11	0.03 ± 0.00^ cd^	0.20 ± 0.01^bd^	2.72 ± 0.16^a^	106.05 ± 2.82^a^	9.13 ± 1.46^bc^	173.57 ± 9.25f.
4P1	0.03 ± 0.00^d^	0.13 ± 0.00^e^	2.42 ± 0.10^a^	110.71 ± 5.39^a^	10.79 ± 0.67^ce^	232.43 ± 4.25^ g^
3P5	0.11 ± 0.00^e^	0.14 ± 0.00^e^	2.33 ± 0.15^a^	141.12 ± 0.44^b^	7.57 ± 0.63^bf^	125.86 ± 1.36^ h^
5P3	0.05 ± 0.00^a^	0.06 ± 0.00f.	7.40 ± 0.64^b^	341.31 ± 2.91^c^	10.47 ± 0.69^ce^	314.91 ± 3.60^i^
YR1	0.10 ± 0.00^e^	0.06 ± 0.00f.	5.85 ± 0.63^c^	178.33 ± 2.91^d^	9.88 ± 0.45^bc^	160.78 ± 2.68^a^

*DPPH* Diphenyl-1-picrylhydrazyl, *ABTS* 2, 2′-Azino-Bis-3-Ethylbenzothiazoline-6-Sulfonic Acid. Different letters in the same column indicate significant differences (p < 0.05) within conditions according to Tukey’s multiple range Test.

Total phenolic content (TPC) in 80% methanolic extracts of the 10 Sorghum ecotypes at a concentration of 1 mg/ml ranged from 125.86 ± 1.36 to 314.91 ± 3.60 mg GAE/g dw. The ecotype 5P3 had a significantly higher TPC (314.91 ± 3.60 mg GAE/g dw) and the lowest TPC was observed in ecotype 3P5. Additionally, a significant (*p* < 0.05) difference in the TPC among the ecotypes was noticed.

The total flavonoid content (TFC) ranged from 4.53 ± 0.91 to 14.04 ± 1.71 mg GAE/g dw for the 10 ecotypes. The highest TFC was observed in ecotype 1P1 (14.04 ± 1.71 mg GAE/g dw), while the lowest TFC (4.53 ± 0.91 mg GAE/g dw) was recorded in extracts of ecotype 3P4. Given that the solvents and standards used in our study are different from other published studies, it is difficult to make direct comparisons between our values and those of literature. Nevertheless, they offer a useful way of characterizing the grain material regarding phenolic content^26^. Other studies have reported TPC and TFC of different ecotypes of sorghum. Awika et al.^39^ reported that the TPC of eight sorghum varieties from Kenya ranged from 7.9 ± 0.10 to 29.6 ± 0.7 (mg GAE/g). In addition, these authors demonstrated that the TPC of crude freeze-dried sorghum extracts of the same varieties ranged from 48.3 ± 0.3 to 308 ± 13 (mg GAE/g). Another study by Rao et al.^9^ indicated that the TPC of six varieties of *Sorghum bicolor* cultivated in Australia ranged from 0.24 ± 0.03 to 11.50 ± 1.81 mg/g GAE. Along the same line, TPC of seven varieties of Senegalese sorghum ranged from 0.22 ± 0.01 to 0.57 ± 0.01 (mg GAE/100 mg of dry matter)^40^. Moreover, Ofosu et al.^41^ reported that the TPC of eight ethanolic extracts of sorghum cultivated in Korea ranged from 122.6 ± 22.0 to 174.4 ± 18.0 (mg ferulic acid equivalent/ 100 g, dw). Regarding TFC, the authors reported that the TFC of ethanolic extracts of these sorghum varieties ranged from 114.0 ± 13.2 to 138.5 ± 10.8 (mg catechin equivalent/100 g, dw)^41^. Using a maceration extraction method, TFC from various Indian sorghum genotypes ranged from 24.26 ± 0.12 to 117.87 ± 0.23 mg QE/g according to the study by Kumari et al.^42^. Moreover, Kim et al.^43^ showed that the TFC in sorghum plants ranged from 14.5 to 38.1 mg QE/g. Whereas, in Devis et al.^44^ study, the TFC ranged from 65 to 142 mg QE/g using an acidified methanol extraction. These variations in TPC and TFC can be observed due to the difference in extraction process, solvents, genotype of the cultivars, and temperature conditions^42^.

DPPH radical scavenging capacity of the extracts of the 10 ecotypes of *Sorghum bicolor* are shown in Table 5. The IC_50_ values of the studied ecotypes ranged from 0.02 ± 0.00 to 0.11 ± 0.00 mg/ml. The extracts from ecotype 1P12 and 3P9 exhibited the highest antioxidant capacity as represented by a lower IC_50_ value (0.02 ± 0.00 mg/ml), while the lowest DPPH radical scavenging activity was observed in the extracts of 3P5 (0.11 ± 0.00 mg/ml).

ABTS radical scavenging capacity of the different ecotypes of *Sorghum bicolor* varied considerably (Table 5). 5P3 and YR1 extracts presented the best results (lowest IC_50_ values) (0.06 ± 0.00 mg/ml), while 1P1 extract presented the lowest results (0.34 ± 0.02 mg/ml). These findings are consistent with earlier reports which demonstrated powerful ABTS and DPPH radical scavenging activities of methanol extracts from red sorghum^45^, acidified methanol extracts from Kenyan varieties (52–112 µmol Trolox equivalents/g (TE/g) for ABTS value)^46^, non-tannin whole-grain varieties from aqueous acetone extracts (63.9–78.9 for ABTS value and 15.3–22.2 µmol TE/g for DPPH)^39^, and tannin whole-grain varieties from aqueous acetone extracts ( 61.6–125 for ABTS and 17.7–44.7 µmol TE/g for DPPH)^45^.

The present study showed that all ecotypes of *Sorghum bicolor* are endowed with metal chelating activity (Table 5). The higher IC_50_ value was obtained for the extract of the 5P3 (IC_50_ = 7.40 ± 0.64 mg/ml), whereas 3P5 had the lowest activity (IC_50_ = 2.33 ± 0.15 mg/ml). In a previous study, Irondi et al.^45^ evaluated the Fe^2+^ chelating activity of raw and roasted red sorghum grains and obtained an IC_50_ of 19.83 ± 1.02 and 17.68 ± 0.94 µg/ml respectively which are low compared to our results. The FRAP assay of the 10 *Sorghum bicolor* ecotypes is presented in Table 5. The reducing power was extremely higher in the 5P3 (341.31 ± 2.91 mg AAE/g dw) compared to the other ecotypes. Ecotype 1P1 had the lowest reducing power (103.15 ± 2.81 mg AAE/g dw). Results from ANOVA analysis revealed that the metal chelating and the FRAP assay results showed non-significant differences (*p* ˃ 0 0.05) among some of the tested sorghum ecotypes (1P1, 1P12, 2P7, 3P4, 3P9, 4P11, and 4P1). Compared to our results, Rao et al.^9^ showed that six varieties of sorghum grown in Australia had a ferric reducing activity that ranged from 2.31 ± 1.55 to 20.92 ± 2.69 mg/g Trolox Equivalent. Similarly, a study by López-Contreras et al.^47^, also revealed that the FRAP value of ten *Sorghum bicolor* genotypes from different geographic origins including Mexico and India ranged from 2.62 ± 0.06 to 98.50 ± 0.25 µmol TE/g. The results obtained from this study indicate a considerable variation for TPC, TFC, and antioxidant activities among the ecotypes which will have a potential interest to plant breeders.

#### Correlation analysis among biochemical traits

Antioxidants are known for their ability to scavenge free radicals (assessed here by DPPH and ABTS scavenging assays) as well as their ability to decrease a higher valent element to its lower valence state (evaluated here by the FRAP and iron chelation assays)^48^. In our study, there was a certain overlap between the antioxidant activities of TPC and TFC from sorghum grains. Indeed, based on the Pearson’s correlation coefficient, our results showed a moderate positive correlation between TFC and ABTS. On the other hand, the same test showed important positive correlation between TPC and FRAP and ion chelation (Table 6). This result indicate that sorghum may contain a wide range of natural compounds that could manifest natural antioxidant capacities through different mechanisms.

**Table 6:** Pearson correlation between antioxidants activities, TPC, and TFC.

		DPPH	ABTS	TFC	TPC	FRAP	Iron chelation
DPPH	Pearson correlation	1	− 0.397*	− 0.110	− 0.560**	0.270	0.281
ABTS		− 0.397*	1	0.154	− 0.312*	− 0.644**	− 0.682**
TFC		− 0.110	0.154	1	0.304	0.056	0.078
TPC		− 0.560**	− 0.312*	0.304	1	0.485**	0.452**
FRAP		0.270	− 0.644**	0.056	0.485**	1	0.899**
Iron chelation		0.281	− 0.682**	0.078	0.452**	0.899**	1

*DPPH* Diphenyl-1-picrylhydrazyl, *ABTS* 2, 2′-Azino-Bis-3-Ethylbenzothiazoline-6-Sulfonic Acid, *TFC* Total Flavonoid content, *TPC* Total phenolic content, *FRAP* Ferric reducing antioxidant power.

*The correlation is significant at the 0.05 level (two-tailed).

**The correlation is significant at the 0.01 (two-tailed) level.

### Conclusion

Besides its importance as an animal feed, sorghum is considered as a staple food for millions of people in Africa and Asia. The grains of sorghum are rich in protein, starch, non-starch polysaccharides, and fatty acids in addition to several phytochemicals such as phenolic compounds and flavonoids, which are well known for a wide range of biological activities (antioxidant, anticancer, antidiabetic…etc.). Despite all these traits, sorghum has not benefited from the same selection program as the other cereals and therefore, joined efforts should be managed to further develop this cereal for human consumption. In our study, we selected 10 Moroccan sorghum ecotypes and compared their agromorphological traits using quantitative and qualitative traits which could contribute to the selection programs of high yielding varieties. Moreover, biochemical parameters related to the high added value of sorghum grains, such as the phenolic and flavonoid contents, and antioxidant capacities, were compared as well. The results obtained showed a high diversity among our sorghum collection. Moreover, the results obtained allowed as to suggest that for any selection program for sorghum, or any other crop, it is preferable to evaluate both agromorphological and biochemical traits. We also suggest that the ecotype, named here 4P1, presents high agronomic performance and is endowed with potent antioxidant activities, possibly due to its high levels of flavonoids and phenolic compounds. Therefore, the results of the present study could be of great interest and constitute an initial framework for the selection programs of high yield sorghum varieties with highly beneficial added value.

## Materials and methods

### Plant material, study site, and experimental set-up

The plant material comprised of 10 sorghum ecotypes that were selected from the collections conserved at the Faculty of Sciences and Technologies of Tangier (FSTT). This core collection is representative of the Moroccan cultivated sorghum varieties, which were sampled based on geographical origin. The study was conducted in Tinghir (17° 25′ N latitude and 78° E longitude and minimum and maximum temperatures of 10 °C and 45 °C with an annual total rainfall of 619 mm), during 2015 summer season. Sowing was done following a randomized complete block design (RCBD) with three replicates and in 8 m rows with a row spacing of spaced 60 cm apart and plant to plant spaced 20 cm. Standard cultivation practices followed locally were adopted. NPK fertilizer was applied at the rate of 100 kg/ha. Data of the morpho-agronomic traits were collected from six randomly selected individual plants for each ecotype. Meanwhile, the collected mature seeds were used for further biochemical experiments. The 25 morpho-agronomic traits that were measured or scored are described in Table 7.

**Table 7:** Morpho-agronomic traits evaluated on 10 Moroccan sorghum ecotypes.

Traits (units)	Abbreviation	Method
Plant height (cm)	PH	Length in cm from base of the stalk to the tip of the panicle at maturity
Width of third leaf	WTF	Width of third leaf from top
Length of third leaf	LTF	Length of third leaf from top
Days to flag leaf appearance	DFL	Number of days from sowing to flag leaf appearance
Width of flag leaf	WFL	Width in cm at widest part of flag leaf
Length of flag leaf	LFL	Length in cm from base of flag leaf to leaf tip
Number of internodes	(NI)	Number of internodes from the base of the plant to the base of panicle
Diameter of third internode (cm)	DTI	Diameter of third internode from top was measured with calipers
Length of third internode (cm)	(LTI)	Length in cm of the third internode from top
Peduncle length (cm)	PL	Length in cm from ligule of flag leaf to base of panicle
Peduncle exertion (cm)	EX	Length of peduncle
Panicle length (cm)	PANL	Length in cm from the first whorl of branches to the tip of the rachis (maturity)
Panicle width (cm)	PW	Diameter at broadest part of panicle in cm
Number of primary branches per panicle	NPBP	The number of ramifications produced from the central axes of panicle
Peduncle shape	PS	These traits were observed according to Harlan and De Wet key (1972)^49^. Briefly, the peduncle shape varies from erect to bent. Panicle compactness varies from loose to compact. Grain covering is the percentage of grain covered by glume. Grain shape can be rounded oval, or elliptic. Grain size is length of the longest axis of seed. Grain and glume color vary from white to brown
Panicle compactness	PC	
Grain covering	GCOV	
Grain shape	GSH	
Grain size	GSI	
Grain colour	GCOL	
Glume colour	GLC	
Days to flowering (days)	NDF	Number of days from planting to flower emergence
Weight of 100 grains (g)	HGW	Weight of 100 grains with grain humidity less than or equal to 12%. Based on random sample of 100-seeds taken four times from the bulked seeds of each experimental unit;
Seedling vigor %	GV	Visual score of seedling growth at 20 day after sowing on a scale of 1 to 5, 1 being low vigour and 5 representing high vigour
Germination percentage %	GP	A seed is considered germinated when radical emerged by about 2 mm in length (Mohammadi, 2009)^50^

### Analysis of biochemical traits

#### Preparation of methanolic extracts

Air-dried powder of seeds from 10 studied sorghum ecotypes (4.5 g) was mixed with 45 mL of 80% methanol and kept in shaking at 250 rpm for 12 h at room temperature in the dark. The resulted solutions were thereafter filtered using whatman filter paper and centrifuged at 6000× g for 10 min. Finally, the supernatants were concentrated by evaporation in an incubator at 37 °C.

#### Total phenolic content

The total phenolic content was determined by the method of the Folin–Ciocalteu, as described by Kabach et al.^51^. Briefly, 400 µL of Folin Ciocalteau’s reagent and 1 mL of sodium carbonate (7%) were added to 100 µL of the methanolic plant extracts (1 mg/ml), and the final volume was brought to 1.6 ml with distilled water. The tubes were kept in the dark for 30 min, then the absorbance was read at 725 nm against a blank. Gallic acid at various concentrations (3.125–100 µg/ml) was used to obtain a calibration curve. Total phenolic content of plant extracts was expressed as milligrams of gallic acid equivalent per gram of dry weight (mg GAE/g dw). All experiments were performed in triplicate.

#### Total flavonoid content

The flavonoid content of different extracts was measured using the aluminum trichloride colorimetric method (AlCl_3_) modified by Ben Mrid et al.^52^. Briefly, 10 µL of potassium acetate (1 M) was mixed with 40 µL of plant extracts (1 mg/ml) and 10 µL of aluminum chloride (10%). Then, 100 µL of methanol 50% was added and the total volume was brought to 400 µL with distilled water. The absorbance was measured at 415 nm against a blank. A calibration curve was made using various concentrations (3.125–100 µg/ml) of quercetin and the flavonoid content of tested samples were expressed as mg quercetin equivalent per gram of dry weight (mg GAE/g dw). All experiments were performed in triplicate.

### Antioxidant activity

#### DPPH radical scavenging assay

Diphenyl-1-picrylhydrazyl (DPPH) assay was conducted to determine the radical scavenging activity of the different extracts as adopted by Hatano et al.^53^ with some modifications suggested by Asraoui et al.^54^. For this purpose, 50 µL of plants extracts at different concentrations were mixed with 150 µL of 0.004% freshly prepared methanol solution of DPPH in a 96-well plate. The absorbance was read at 517 nm after 30 min of incubation in the dark. The remaining DPPH % was calculated using the formula:

1 $${\text{Total}}\;{\text{antioxidant}}\;{\text{activity }}\left( \% \right) \, = \, \left[ {\left( {{\text{A}}\;{\text{control }}{-}{\text{ A}}\;{\text{sample}}} \right)/{\text{A}}\;{\text{control}}} \right] \, \times { 1}00$$

The IC_50_ value, which represents the concentration of extracts needed to scavenge 50% DPPH free radicals was calculated from the plot of inhibition (%) against the sample extract concentration.

#### *ABTS*^*·*+^*radical scavenging assay*

ABTS^·+^ radical scavenging activity was evaluated following the method of Re et al.^55^. In this assay, ABTS^·+^ was generated by the oxidation of ABTS with potassium persulfate. Prior to assaying, the ABTS^·+^ stock solution was diluted with methanol until it reached an absorbance of 0.700 ± 0.020 at 734 nm. Then 185 µL of the diluted ABTS^·+^ solution was mixed with 15 µL of the plant extracts and the absorbance was measured at 734 nm after 10 min. The ABTS^·+^ scavenging % was calculated as follows:

2 $${\text{ABTS}}^{\cdot + } \;{\text{scavenging }}\% \, = \, \left( {{\text{AB}} - {\text{AA }}/{\text{ AB}}} \right) \, \times { 1}00$$

where AB is the absorbance of ABTS^·+^ and methanol; AA is the absorbance of ABTS^·+^ and the plants extracts.

#### Metal chelating activity

The ferrous ion chelating activity was determined by measuring the formation of the Fe^2+^-ferrozine complex according to Dinis et al.^56^ with slight modifications. Samples of plants extracts (800µL) were mixed with 10 µL of FeCl_2_ (0.6 mM). After 10 min of incubation at room temperature, 50 µL of ferrozine (5 mM) was added and the final volume was made up to 1 mL with distilled water. The absorbance was measured at 562 nm after 10 min of incubation. The concentration of the samples required to chelate 50% of Fe^2+^ ions (IC_50_) was calculated from linear regression analysis.

#### Ferric reducing power assay (FRAP)

The ferric reducing power (FRAP) of the extracts was determined according to the method of Oyaizu^57^ with some modifications. Various concentrations (0.06–1 mg/mL) of extracts (200µL) were combined with 500 µL of phosphate buffer (0.2 M, pH 6.6) and 500 µL of 1% potassium ferricyanide (K_3_Fe[CN]_6_). This mixture was incubated at 50 °C for 20 min and then 500 µL of 10% trichloroacetic was added and centrifuged at 3000 rpm for 10 min. The upper layer fraction (500µL) was mixed with 500 µl of distilled water and 100 µl of ferric chloride (FeCl_3_, 0.1%). The absorbance was measured at 700 nm. Results were presented as mg of ascorbic acid equivalent per g of dry weight (mg AAE/g dw).

### Statistical analysis

The statistical software R (version 3.6.3) was used to analyze all the morphological and biochemical traits. Descriptive statistics (i.e., mean, standard deviation…etc.) and analyses of variance (ANOVA) were performed to test significance differences between cultivars for all traits. Tukey’s test at a significance level of *p*<0.05 was carried out for pairwise comparisons of means. Pearson’s correlation coefficient was used to measure correlations between traits using the “cor” function (part of the standard R platform). To identify the patterns of morphological variation, principal component analysis (PCA) was conducted on correlation matrix using “FactoMineR” package. These were used to build the dendogram from Hierarchical Clustering on Principal Components (HCPC) using the same package (FactoMineR).

### Human and animal rights

We have received permission from FSTT for conducting research on 10 sorghum ecotypes selected from FSTT’s core collection of the Moroccan cultivated sorghum varieties. The use of plants and the research experimental protocol in the present study are complied with international, national and/or institutional guidelines and legislation. This study does not contain any studies with human participants or animals performed by any of the authors.

## Data Availability

The datasets used and/or analyzed during the current study are available from the corresponding author on reasonable request.
